# 9-(2,5-Dimethyl­phen­oxy­carbon­yl)-10-methyl­acridinium trifluoro­methane­sulfonate

**DOI:** 10.1107/S1600536811045090

**Published:** 2011-11-05

**Authors:** Damian Trzybiński, Karol Krzymiński, Jerzy Błażejowski

**Affiliations:** a Faculty of Chemistry, University of Gdańsk, J. Sobieskiego 18, 80-952 Gdańsk, Poland

## Abstract

In the title compound, C_23_H_20_NO_2_^+^·CF_3_SO_3_^−^, the acridine ring system is oriented at a dihedral angle of 23.1 (1)° with respect to the benzene ring and the carboxyl group is twisted at an angle of 74.1 (1)° relative to the acridine skeleton. In the crystal, adjacent cations are linked through C—H⋯π inter­actions and neighboring cations and anions *via* weak C—H⋯O hydrogen bonds. The mean planes of adjacent acridine units are either parallel or inclined at angles of 15.0 (1), 26.9 (1) and 48.1 (1)° in the crystal structure.

## Related literature

For general background to the chemiluminogenic properties of 9-phen­oxy­carbonyl-10-methyl­acridinium trifluoro­meth­ane­­sulfonates, see: Brown *et al.* (2009 ▶); King *et al.* (2007 ▶); Krzymiński *et al.* (2011 ▶); Roda *et al.* (2003 ▶). For related structures, see: Krzymiński *et al.* (2009 ▶). For inter­molecular inter­actions, see: Novoa *et al.* (2006 ▶); Takahashi *et al.* (2001 ▶). For the synthesis, see: Sato (1996 ▶); Krzymiński *et al.* (2011 ▶).
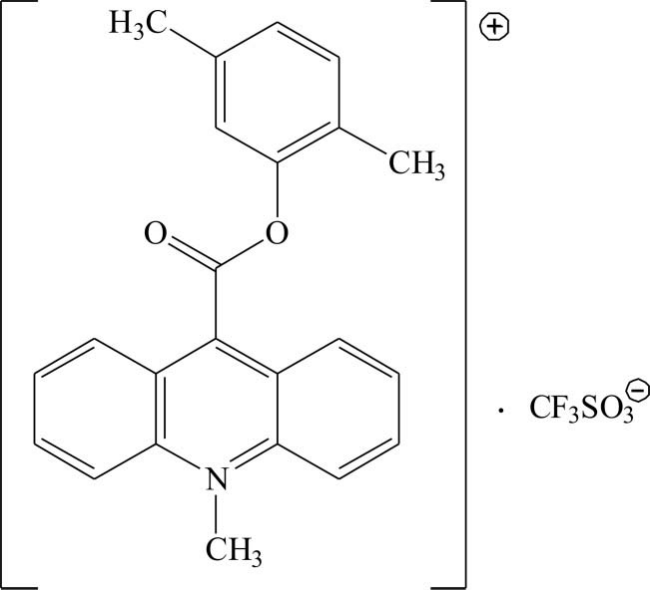


## Experimental

### 

#### Crystal data

C_23_H_20_NO_2_^+^·CF_3_SO_3_^−^
*M* *_r_* = 491.48
Orthorhombic, 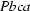

*a* = 12.3604 (17) Å
*b* = 17.341 (3) Å
*c* = 21.101 (3) Å
*V* = 4522.8 (12) Å^3^
*Z* = 8
Mo *K*α radiation
μ = 0.21 mm^−1^
*T* = 295 K
0.60 × 0.15 × 0.10 mm

#### Data collection

Oxford Diffraction Gemini R Ultra Ruby CCD diffractometer
Absorption correction: multi-scan (*CrysAlis RED*; Oxford Diffraction, 2008 ▶) *T*_min_ = 0.960, *T*_max_ = 0.985
32535 measured reflections
4000 independent reflections
2050 reflections with *I* > 2σ(*I*)
*R*_int_ = 0.106

#### Refinement

*R*[*F* ^2^ > 2σ(*F* ^2^)] = 0.061
*wR*(*F* ^2^) = 0.185
*S* = 1.01
4000 reflections
310 parameters
H-atom parameters constrained
Δρ_max_ = 0.36 e Å^−3^
Δρ_min_ = −0.23 e Å^−3^

### 

Data collection: *CrysAlis CCD* (Oxford Diffraction, 2008 ▶); cell refinement: *CrysAlis RED* (Oxford Diffraction, 2008 ▶); data reduction: *CrysAlis RED*; program(s) used to solve structure: *SHELXS97* (Sheldrick, 2008 ▶); program(s) used to refine structure: *SHELXL97* (Sheldrick, 2008 ▶); molecular graphics: *ORTEP-3* (Farrugia, 1997 ▶); software used to prepare material for publication: *SHELXL97* and *PLATON* (Spek, 2009 ▶).

## Supplementary Material

Crystal structure: contains datablock(s) global, I. DOI: 10.1107/S1600536811045090/xu5358sup1.cif

Structure factors: contains datablock(s) I. DOI: 10.1107/S1600536811045090/xu5358Isup2.hkl

Supplementary material file. DOI: 10.1107/S1600536811045090/xu5358Isup3.cml

Additional supplementary materials:  crystallographic information; 3D view; checkCIF report

## Figures and Tables

**Table 1:** Hydrogen-bond geometry (Å, °) *Cg*2 is the centroid of the C1–C4/C11/C12 benzene ring.

*D*—H⋯*A*	*D*—H	H⋯*A*	*D*⋯*A*	*D*—H⋯*A*
C4—H4⋯O29^i^	0.93	2.59	3.257 (5)	129
C5—H5⋯O30	0.93	2.58	3.466 (5)	160
C6—H6⋯O28	0.93	2.52	3.303 (5)	142
C7—H7⋯O29^ii^	0.93	2.39	3.188 (5)	144
C20—H20⋯*Cg*2^iii^	0.93	2.81	3.439 (4)	126
C25—H25*B*⋯O28^ii^	0.96	2.49	3.289 (5)	141
C26—H26*A*⋯O30^i^	0.96	2.47	3.314 (5)	146

Symmetry codes: (i) 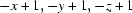; (ii) 
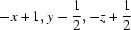
; (iii) 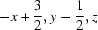.

**Fig. 1.**
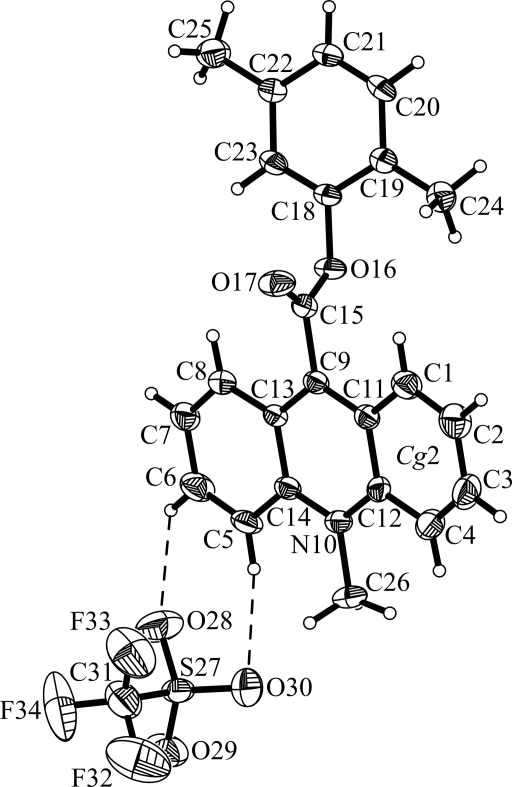
The molecular structure of the title compound showing the atom-labeling scheme. Displacement ellipsoids are drawn at the 25% probability level and H atoms are shown as small spheres of arbitrary radius. Cg2 denotes the ring centroid. The C–H···O interactions are represented by dashed lines.

**Fig. 2.**
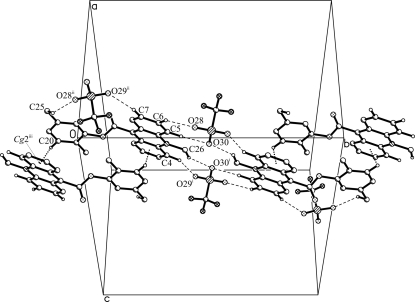
The arrangement of the ions in the crystal structure. The C–H···O interactions are represented by dashed lines, the C–H···π contacts by dotted lines. H atoms not involved in interactions have been omitted. [Symmetry codes: (i) –x + 1, –y + 1, –z + 1; (ii) –x + 1, y – 1/2, –z + 1/2; (iii) –x + 3/2, y – 1/2, z.]

## supplementary crystallographic information

### Comment

Chemiluminescing 9-(phenoxycarbonyl)-10-methylacridinium cations are widely applied as indicators or fragments of labels in assays of biologically and environmentally important entities such as antigens, antibodies, enzymes or DNA fragments (Roda *et al.*, 2003; King *et al.*, 2007; Brown *et al.*, 2009). The cations of these salts are oxidized by H_2_O_2_ in alkaline media, a reaction that is accompanied by the removal of the phenoxycarbonyl fragment and the conversion of the remaining part of the molecules to electronically excited, light-emitting 10-methyl-9-acridinone (Krzymiński *et al.*, 2011). The efficiency of chemiluminescence – crucial to analytical applications – is affected by the constitution of the phenyl fragment. Here we present the crystal structure of 9-(2,5-dimethylphenoxycarbonyl)-10-methylacridinium trifluoromethanesulfonate, whose chemiluminogenic features we have thoroughly investigated (Krzymiński *et al.*, 2011).

In the cation of the title compound (Fig. 1), the bond lengths and angles characterizing the geometry of the acridinium moiety are typical of acridine-based derivatives (Krzymiński *et al.*, 2009). With respective average deviations from planarity of 0.022 (3) Å and 0.006 (3) Å, the acridine and benzene ring systems are oriented at a dihedral angle of 23.1 (1)°. The carboxyl group is twisted at an angle of 74.1 (1)° relative to the acridine skeleton. The mean planes of the adjacent acridine moieties are parallel (at an angle 0.0 (1)°) or inclined at angles of 15.0 (1), 26.9 (1) and 48.1 (1)° in the crystal lattice.

In the crystal structure, the adjacent cations are linked by C–H···π (Table 1, Fig. 2) contacts and the neighboring cations and anions via C–H···O (Table 1, Figs. 1 and 2) interactions. The C–H···O interactions are of the hydrogen bond type (Novoa *et al.* 2006), while the C–H···π (Takahashi *et al.*, 2001) contacts should be of an attractive nature. The crystal structure is stabilized by a network of these specific short-range interactions and by long-range electrostatic interactions between ions.

### Experimental

2,5-Dimethylphenylacridine-9-carboxylate was synthesized by esterification of 9-(chlorocarbonyl)acridine (obtained in the reaction of acridine-9-carboxylic acid with a tenfold molar excess of thionyl chloride) with 2,5-dimethylphenol in anhydrous dichloromethane in the presence of N,N-diethylethanamine and a catalytic amount of N,N-dimethyl-4-pyridinamine (room temperature, 15 h) (Sato, 1996; Krzymiński *et al.*, 2011). The product was purified chromatographically (SiO_2_, cyclohexane/ethyl acetate, 1/1 v/v) and subsequently quaternarized with a fivefold molar excess of methyl trifluoromethanesulfonate dissolved in anhydrous dichloromethane. The crude 9-(2,5-dimethylphenoxycarbonyl)-10-methylacridinium trifluoromethanesulfonate was dissolved in a small amount of ethanol, filtered and precipitated with a 20 v/v excess of diethyl ether. Yellow crystals suitable for X-ray investigations were grown from ethanol/water solution (1:1 v/v) (m.p. 509–511 K).

### Refinement

H atoms were positioned geometrically, with C—H = 0.93 Å and 0.96 Å for the aromatic and methyl H atoms, respectively, and constrained to ride on their parent atoms with *U*_iso_(H) = *xU*_eq_(C), where *x* = 1.2 for the aromatic and *x* = 1.5 for the methyl H atoms.

### Crystal data

**Table:**

C_23_H_20_NO_2_^+^·CF_3_SO_3_^−^	*F*(000) = 2032
*M**_r_* = 491.48	*D*_x_ = 1.444 Mg m^−^^3^
Orthorhombic, *P**b**c**a*	Mo *K*α radiation, λ = 0.71073 Å
Hall symbol: -P 2ac 2ab	Cell parameters from 4734 reflections
*a* = 12.3604 (17) Å	θ = 3.4–26.0°
*b* = 17.341 (3) Å	µ = 0.21 mm^−^^1^
*c* = 21.101 (3) Å	*T* = 295 K
*V* = 4522.8 (12) Å^3^	Needle, yellow
*Z* = 8	0.60 × 0.15 × 0.10 mm

### Data collection

**Table:**

Oxford Diffraction Gemini R Ultra Ruby CCD diffractometer	4000 independent reflections
Radiation source: enhanced (Mo) X-ray source	2050 reflections with *I* > 2σ(*I*)
graphite	*R*_int_ = 0.106
Detector resolution: 10.4002 pixels mm^-1^	θ_max_ = 25.1°, θ_min_ = 3.5°
ω scans	*h* = −14→12
Absorption correction: multi-scan (*CrysAlis RED*; Oxford Diffraction, 2008)	*k* = −20→20
*T*_min_ = 0.960, *T*_max_ = 0.985	*l* = −23→25
32535 measured reflections	

### Refinement

**Table:**

Refinement on *F*^2^	Primary atom site location: structure-invariant direct methods
Least-squares matrix: full	Secondary atom site location: difference Fourier map
*R*[*F*^2^ > 2σ(*F*^2^)] = 0.061	Hydrogen site location: inferred from neighbouring sites
*wR*(*F*^2^) = 0.185	H-atom parameters constrained
*S* = 1.01	*w* = 1/[σ^2^(*F*_o_^2^) + (0.0901*P*)^2^ + 0.2728*P*] where *P* = (*F*_o_^2^ + 2*F*_c_^2^)/3
4000 reflections	(Δ/σ)_max_ < 0.001
310 parameters	Δρ_max_ = 0.36 e Å^−^^3^
0 restraints	Δρ_min_ = −0.23 e Å^−^^3^

### Special details

**Table:**

Geometry. All esds (except the esd in the dihedral angle between two l.s. planes) are estimated using the full covariance matrix. The cell esds are taken into account individually in the estimation of esds in distances, angles and torsion angles; correlations between esds in cell parameters are only used when they are defined by crystal symmetry. An approximate (isotropic) treatment of cell esds is used for estimating esds involving l.s. planes.

### Fractional atomic coordinates and isotropic or equivalent isotropic displacement parameters (Å^2^)

**Table:**

	*x*	*y*	*z*	*U*_iso_*/*U*_eq_	
C1	0.7203 (3)	0.1485 (2)	0.5953 (2)	0.0692 (11)	
H1	0.7452	0.0981	0.5906	0.083*	
C2	0.7290 (4)	0.1843 (3)	0.6520 (2)	0.0872 (14)	
H2	0.7599	0.1587	0.6863	0.105*	
C3	0.6911 (4)	0.2608 (3)	0.6593 (2)	0.0858 (14)	
H3	0.6977	0.2849	0.6984	0.103*	
C4	0.6455 (3)	0.2995 (2)	0.6105 (2)	0.0713 (12)	
H4	0.6197	0.3493	0.6167	0.086*	
C5	0.5462 (3)	0.3125 (2)	0.3893 (2)	0.0667 (11)	
H5	0.5235	0.3633	0.3943	0.080*	
C6	0.5383 (4)	0.2782 (2)	0.3321 (2)	0.0815 (13)	
H6	0.5102	0.3061	0.2981	0.098*	
C7	0.5711 (4)	0.2021 (2)	0.3224 (2)	0.0720 (12)	
H7	0.5637	0.1796	0.2826	0.086*	
C8	0.6132 (3)	0.1613 (2)	0.3704 (2)	0.0600 (10)	
H8	0.6365	0.1111	0.3633	0.072*	
C9	0.6641 (3)	0.15331 (18)	0.48358 (18)	0.0474 (9)	
N10	0.5964 (2)	0.30431 (15)	0.50005 (16)	0.0503 (7)	
C11	0.6738 (3)	0.18708 (19)	0.54298 (17)	0.0502 (9)	
C12	0.6370 (3)	0.26519 (19)	0.55098 (19)	0.0503 (9)	
C13	0.6231 (2)	0.19343 (17)	0.43212 (17)	0.0452 (9)	
C14	0.5886 (3)	0.27166 (18)	0.44149 (19)	0.0493 (9)	
C15	0.7004 (3)	0.07054 (19)	0.47513 (17)	0.0504 (9)	
O16	0.61431 (18)	0.02474 (12)	0.47079 (13)	0.0576 (7)	
O17	0.7921 (2)	0.05008 (14)	0.47341 (14)	0.0755 (9)	
C18	0.6283 (3)	−0.05622 (18)	0.46459 (19)	0.0483 (9)	
C19	0.6315 (3)	−0.09995 (19)	0.51964 (19)	0.0518 (9)	
C20	0.6311 (2)	−0.1796 (2)	0.5100 (2)	0.0563 (10)	
H20	0.6318	−0.2121	0.5451	0.068*	
C21	0.6297 (3)	−0.2116 (2)	0.4508 (2)	0.0552 (10)	
H21	0.6294	−0.2650	0.4468	0.066*	
C22	0.6289 (3)	−0.16674 (19)	0.39689 (19)	0.0542 (10)	
C23	0.6272 (3)	−0.08684 (19)	0.4050 (2)	0.0553 (10)	
H23	0.6252	−0.0545	0.3699	0.066*	
C24	0.6348 (3)	−0.0649 (2)	0.5843 (2)	0.0699 (11)	
H24A	0.5841	−0.0231	0.5865	0.105*	
H24B	0.7063	−0.0459	0.5927	0.105*	
H24C	0.6161	−0.1032	0.6153	0.105*	
C25	0.6290 (4)	−0.2022 (2)	0.3319 (2)	0.0846 (13)	
H25A	0.7006	−0.1995	0.3143	0.127*	
H25B	0.5796	−0.1746	0.3051	0.127*	
H25C	0.6070	−0.2552	0.3348	0.127*	
C26	0.5592 (3)	0.38590 (18)	0.5077 (2)	0.0727 (12)	
H26A	0.5623	0.4000	0.5517	0.109*	
H26B	0.4862	0.3906	0.4927	0.109*	
H26C	0.6054	0.4194	0.4836	0.109*	
S27	0.44245 (10)	0.50390 (5)	0.30301 (6)	0.0740 (4)	
O28	0.4254 (3)	0.43431 (19)	0.2715 (2)	0.1310 (14)	
O29	0.3900 (3)	0.57019 (19)	0.2803 (2)	0.1239 (13)	
O30	0.4320 (3)	0.4936 (2)	0.37051 (17)	0.1162 (12)	
C31	0.5832 (5)	0.5223 (4)	0.2920 (3)	0.1130 (19)	
F32	0.6101 (4)	0.5876 (3)	0.3223 (3)	0.218 (3)	
F33	0.6458 (3)	0.4707 (3)	0.3148 (2)	0.1741 (18)	
F34	0.6094 (4)	0.5364 (4)	0.2353 (3)	0.221 (3)	

### Atomic displacement parameters (Å^2^)

**Table:**

	*U*^11^	*U*^22^	*U*^33^	*U*^12^	*U*^13^	*U*^23^
C1	0.084 (3)	0.060 (2)	0.064 (3)	0.000 (2)	−0.005 (2)	0.006 (2)
C2	0.113 (4)	0.092 (4)	0.056 (3)	0.000 (3)	−0.015 (3)	0.008 (3)
C3	0.103 (3)	0.097 (4)	0.058 (3)	−0.004 (3)	0.010 (3)	−0.017 (3)
C4	0.074 (3)	0.072 (3)	0.068 (3)	0.001 (2)	0.009 (2)	−0.011 (3)
C5	0.085 (3)	0.039 (2)	0.076 (3)	0.0017 (18)	−0.002 (2)	0.014 (2)
C6	0.115 (4)	0.061 (3)	0.069 (3)	0.000 (2)	−0.019 (3)	0.016 (3)
C7	0.107 (3)	0.059 (3)	0.050 (3)	−0.010 (2)	−0.005 (2)	0.002 (2)
C8	0.076 (3)	0.044 (2)	0.061 (3)	−0.0047 (17)	0.006 (2)	−0.002 (2)
C9	0.0457 (18)	0.0386 (18)	0.058 (3)	−0.0028 (14)	0.0055 (17)	0.0033 (18)
N10	0.0522 (16)	0.0377 (15)	0.061 (2)	−0.0010 (12)	0.0015 (15)	−0.0024 (16)
C11	0.057 (2)	0.045 (2)	0.049 (2)	−0.0060 (16)	0.0012 (18)	0.0022 (19)
C12	0.0508 (19)	0.049 (2)	0.051 (3)	−0.0053 (16)	0.0118 (17)	−0.005 (2)
C13	0.0497 (19)	0.0353 (18)	0.051 (2)	−0.0035 (14)	0.0058 (16)	0.0035 (18)
C14	0.052 (2)	0.0384 (18)	0.057 (3)	−0.0064 (15)	0.0017 (18)	0.0023 (19)
C15	0.054 (2)	0.0421 (19)	0.056 (3)	0.0008 (17)	0.0040 (18)	0.0039 (17)
O16	0.0504 (14)	0.0342 (12)	0.088 (2)	0.0021 (10)	0.0011 (13)	0.0011 (13)
O17	0.0518 (16)	0.0522 (15)	0.123 (3)	0.0057 (12)	0.0045 (16)	−0.0069 (16)
C18	0.0467 (19)	0.0346 (18)	0.064 (3)	0.0009 (14)	−0.0008 (18)	0.0045 (19)
C19	0.0448 (19)	0.048 (2)	0.062 (3)	−0.0001 (15)	−0.0006 (18)	0.005 (2)
C20	0.050 (2)	0.047 (2)	0.072 (3)	−0.0021 (16)	−0.0019 (19)	0.023 (2)
C21	0.051 (2)	0.0379 (19)	0.076 (3)	−0.0014 (15)	−0.0022 (19)	0.005 (2)
C22	0.058 (2)	0.044 (2)	0.061 (3)	0.0023 (16)	−0.0029 (19)	0.003 (2)
C23	0.061 (2)	0.042 (2)	0.062 (3)	0.0027 (16)	−0.0010 (19)	0.014 (2)
C24	0.075 (3)	0.071 (3)	0.064 (3)	−0.001 (2)	0.001 (2)	0.003 (2)
C25	0.117 (4)	0.064 (3)	0.073 (3)	0.004 (2)	−0.008 (3)	−0.004 (2)
C26	0.087 (3)	0.040 (2)	0.092 (3)	0.0105 (19)	0.001 (2)	−0.013 (2)
S27	0.1049 (9)	0.0443 (6)	0.0728 (9)	0.0084 (5)	−0.0034 (6)	−0.0028 (6)
O28	0.172 (4)	0.080 (2)	0.141 (4)	0.003 (2)	−0.026 (3)	−0.040 (2)
O29	0.140 (3)	0.080 (2)	0.152 (4)	0.027 (2)	−0.003 (3)	0.034 (2)
O30	0.166 (4)	0.112 (3)	0.071 (2)	0.007 (2)	0.020 (2)	0.007 (2)
C31	0.130 (5)	0.102 (4)	0.108 (5)	−0.007 (4)	0.007 (4)	0.029 (4)
F32	0.187 (4)	0.143 (4)	0.323 (8)	−0.075 (3)	−0.048 (4)	0.026 (4)
F33	0.110 (3)	0.186 (4)	0.227 (5)	0.038 (2)	0.005 (2)	0.091 (3)
F34	0.167 (4)	0.357 (7)	0.139 (4)	0.025 (4)	0.059 (3)	0.111 (5)

### Geometric parameters (Å, °)

**Table:**

C1—C2	1.354 (6)	O16—C18	1.421 (4)
C1—C11	1.413 (5)	C18—C23	1.364 (5)
C1—H1	0.9300	C18—C19	1.388 (5)
C2—C3	1.415 (6)	C19—C20	1.397 (5)
C2—H2	0.9300	C19—C24	1.495 (5)
C3—C4	1.352 (6)	C20—C21	1.367 (5)
C3—H3	0.9300	C20—H20	0.9300
C4—C12	1.394 (5)	C21—C22	1.378 (5)
C4—H4	0.9300	C21—H21	0.9300
C5—C6	1.349 (6)	C22—C23	1.396 (5)
C5—C14	1.411 (5)	C22—C25	1.503 (6)
C5—H5	0.9300	C23—H23	0.9300
C6—C7	1.397 (6)	C24—H24A	0.9600
C6—H6	0.9300	C24—H24B	0.9600
C7—C8	1.340 (5)	C24—H24C	0.9600
C7—H7	0.9300	C25—H25A	0.9600
C8—C13	1.421 (5)	C25—H25B	0.9600
C8—H8	0.9300	C25—H25C	0.9600
C9—C13	1.386 (5)	C26—H26A	0.9600
C9—C11	1.389 (5)	C26—H26B	0.9600
C9—C15	1.514 (5)	C26—H26C	0.9600
N10—C14	1.363 (4)	S27—O28	1.394 (3)
N10—C12	1.366 (4)	S27—O29	1.404 (3)
N10—C26	1.496 (4)	S27—O30	1.441 (4)
C11—C12	1.439 (5)	S27—C31	1.784 (7)
C13—C14	1.436 (4)	C31—F34	1.264 (6)
C15—O17	1.188 (4)	C31—F33	1.276 (6)
C15—O16	1.331 (4)	C31—F32	1.343 (7)
			
C2—C1—C11	120.4 (4)	C23—C18—O16	117.9 (3)
C2—C1—H1	119.8	C19—C18—O16	117.8 (3)
C11—C1—H1	119.8	C18—C19—C20	114.7 (4)
C1—C2—C3	120.0 (4)	C18—C19—C24	122.9 (3)
C1—C2—H2	120.0	C20—C19—C24	122.4 (4)
C3—C2—H2	120.0	C21—C20—C19	122.3 (4)
C4—C3—C2	121.4 (4)	C21—C20—H20	118.8
C4—C3—H3	119.3	C19—C20—H20	118.8
C2—C3—H3	119.3	C20—C21—C22	121.7 (3)
C3—C4—C12	120.4 (4)	C20—C21—H21	119.2
C3—C4—H4	119.8	C22—C21—H21	119.2
C12—C4—H4	119.8	C21—C22—C23	117.3 (4)
C6—C5—C14	120.3 (4)	C21—C22—C25	121.4 (3)
C6—C5—H5	119.8	C23—C22—C25	121.2 (4)
C14—C5—H5	119.8	C18—C23—C22	120.0 (3)
C5—C6—C7	121.7 (4)	C18—C23—H23	120.0
C5—C6—H6	119.1	C22—C23—H23	120.0
C7—C6—H6	119.1	C19—C24—H24A	109.5
C8—C7—C6	120.1 (4)	C19—C24—H24B	109.5
C8—C7—H7	120.0	H24A—C24—H24B	109.5
C6—C7—H7	120.0	C19—C24—H24C	109.5
C7—C8—C13	121.3 (3)	H24A—C24—H24C	109.5
C7—C8—H8	119.3	H24B—C24—H24C	109.5
C13—C8—H8	119.3	C22—C25—H25A	109.5
C13—C9—C11	121.8 (3)	C22—C25—H25B	109.5
C13—C9—C15	119.5 (3)	H25A—C25—H25B	109.5
C11—C9—C15	118.7 (3)	C22—C25—H25C	109.5
C14—N10—C12	122.2 (3)	H25A—C25—H25C	109.5
C14—N10—C26	118.0 (3)	H25B—C25—H25C	109.5
C12—N10—C26	119.8 (3)	N10—C26—H26A	109.5
C9—C11—C1	122.7 (3)	N10—C26—H26B	109.5
C9—C11—C12	118.4 (3)	H26A—C26—H26B	109.5
C1—C11—C12	118.9 (3)	N10—C26—H26C	109.5
N10—C12—C4	121.6 (3)	H26A—C26—H26C	109.5
N10—C12—C11	119.4 (3)	H26B—C26—H26C	109.5
C4—C12—C11	118.9 (4)	O28—S27—O29	118.4 (3)
C9—C13—C8	123.5 (3)	O28—S27—O30	110.5 (2)
C9—C13—C14	118.4 (3)	O29—S27—O30	113.4 (2)
C8—C13—C14	118.1 (3)	O28—S27—C31	103.9 (3)
N10—C14—C5	121.7 (3)	O29—S27—C31	105.1 (3)
N10—C14—C13	119.8 (3)	O30—S27—C31	103.8 (3)
C5—C14—C13	118.5 (3)	F34—C31—F33	109.8 (6)
O17—C15—O16	125.6 (3)	F34—C31—F32	102.9 (6)
O17—C15—C9	124.7 (3)	F33—C31—F32	105.2 (6)
O16—C15—C9	109.7 (3)	F34—C31—S27	114.0 (5)
C15—O16—C18	119.9 (2)	F33—C31—S27	114.6 (5)
C23—C18—C19	124.0 (3)	F32—C31—S27	109.3 (5)
			
C11—C1—C2—C3	0.1 (6)	C9—C13—C14—N10	−0.3 (4)
C1—C2—C3—C4	0.2 (7)	C8—C13—C14—N10	179.7 (3)
C2—C3—C4—C12	−1.5 (7)	C9—C13—C14—C5	−179.6 (3)
C14—C5—C6—C7	0.1 (6)	C8—C13—C14—C5	0.4 (5)
C5—C6—C7—C8	−1.0 (7)	C13—C9—C15—O17	105.8 (4)
C6—C7—C8—C13	1.6 (6)	C11—C9—C15—O17	−73.8 (5)
C13—C9—C11—C1	−176.8 (3)	C13—C9—C15—O16	−74.9 (4)
C15—C9—C11—C1	2.8 (5)	C11—C9—C15—O16	105.5 (3)
C13—C9—C11—C12	1.7 (5)	O17—C15—O16—C18	1.2 (6)
C15—C9—C11—C12	−178.7 (3)	C9—C15—O16—C18	−178.1 (3)
C2—C1—C11—C9	179.2 (4)	C15—O16—C18—C23	−95.3 (4)
C2—C1—C11—C12	0.7 (5)	C15—O16—C18—C19	91.0 (4)
C14—N10—C12—C4	179.4 (3)	C23—C18—C19—C20	−1.3 (5)
C26—N10—C12—C4	−0.3 (5)	O16—C18—C19—C20	172.0 (3)
C14—N10—C12—C11	0.4 (5)	C23—C18—C19—C24	178.8 (3)
C26—N10—C12—C11	−179.2 (3)	O16—C18—C19—C24	−7.9 (5)
C3—C4—C12—N10	−176.6 (4)	C18—C19—C20—C21	1.1 (5)
C3—C4—C12—C11	2.3 (5)	C24—C19—C20—C21	−178.9 (3)
C9—C11—C12—N10	−1.5 (5)	C19—C20—C21—C22	0.2 (5)
C1—C11—C12—N10	177.0 (3)	C20—C21—C22—C23	−1.4 (5)
C9—C11—C12—C4	179.5 (3)	C20—C21—C22—C25	179.1 (3)
C1—C11—C12—C4	−1.9 (5)	C19—C18—C23—C22	0.1 (5)
C11—C9—C13—C8	179.1 (3)	O16—C18—C23—C22	−173.1 (3)
C15—C9—C13—C8	−0.4 (5)	C21—C22—C23—C18	1.2 (5)
C11—C9—C13—C14	−0.8 (5)	C25—C22—C23—C18	−179.3 (3)
C15—C9—C13—C14	179.6 (3)	O28—S27—C31—F34	67.3 (6)
C7—C8—C13—C9	178.7 (3)	O29—S27—C31—F34	−57.8 (6)
C7—C8—C13—C14	−1.3 (5)	O30—S27—C31—F34	−177.1 (6)
C12—N10—C14—C5	179.8 (3)	O28—S27—C31—F33	−60.5 (6)
C26—N10—C14—C5	−0.5 (5)	O29—S27—C31—F33	174.5 (5)
C12—N10—C14—C13	0.5 (4)	O30—S27—C31—F33	55.1 (6)
C26—N10—C14—C13	−179.8 (3)	O28—S27—C31—F32	−178.2 (5)
C6—C5—C14—N10	−179.2 (4)	O29—S27—C31—F32	56.7 (5)
C6—C5—C14—C13	0.2 (5)	O30—S27—C31—F32	−62.6 (5)

### Hydrogen-bond geometry (Å, °)

**Table:**

*Cg*2 is the centroid of the C1–C4/C11/C12 benzene ring.

**Table:**

*D*—H···*A*	*D*—H	H···*A*	*D*···*A*	*D*—H···*A*
C4—H4···O29^i^	0.93	2.59	3.257 (5)	129
C5—H5···O30	0.93	2.58	3.466 (5)	160
C6—H6···O28	0.93	2.52	3.303 (5)	142
C7—H7···O29^ii^	0.93	2.39	3.188 (5)	144
C20—H20···Cg2^iii^	0.93	2.81	3.439 (4)	126
C25—H25B···O28^ii^	0.96	2.49	3.289 (5)	141
C26—H26A···O30^i^	0.96	2.47	3.314 (5)	146

Symmetry codes:  (i) −*x*+1, −*y*+1, −*z*+1; (ii) −*x*+1, *y*−1/2, −*z*+1/2; (iii) −*x*+3/2, *y*−1/2, *z*.
